# Determining the Optimal Virtual Reality Exergame Approach for Balance Therapy in Persons With Neurological Disorders Using a Rasch Analysis: Longitudinal Observational Study

**DOI:** 10.2196/30366

**Published:** 2022-03-22

**Authors:** Evelyne Wiskerke, Jan Kool, Roger Hilfiker, Karl Martin Sattelmayer, Geert Verheyden

**Affiliations:** 1 Department of Rehabilitation Sciences KU Leuven - University of Leuven Leuven Belgium; 2 Rehazentrum Valens Kliniken Valens Valens Switzerland; 3 School of Health Sciences HES-SO Valais-Wallis Leukerbad Switzerland

**Keywords:** digital therapeutics, virtual reality, exergaming, balance, stroke, multiple sclerosis, neurorehabilitation, Rasch analysis

## Abstract

**Background:**

Virtual reality (VR) exergames have gained popularity in the rehabilitation of persons with neurological disorders as an add-on therapy to increase intensity of training. Intensity is strongly dependent on the motivation of the patient. Motivation can be increased by delivering variation within training and challenging exercises. However, patients are often underchallenged, as exergame difficulty often does not match the patient’s ability. A Rasch analysis can establish hierarchy of exergame items in order to assist the delivery of patient-centered therapy.

**Objective:**

The aim of this study was to apply the Rasch model to create a hierarchical order of existing VR balance exergames and to relate these exergames to the abilities of persons with neurological disorders, in order to deliver challenge and variation.

**Methods:**

A total of 30 persons with stroke and 51 persons with multiple sclerosis (MS) were included in the study. All participants performed a training program, lasting 3 weeks for persons with MS and 4 weeks for persons with stroke, in which they performed VR balance exergames with a movement recognition–based system (MindMotion GO; MindMaze SA). VR exercise scores, Berg Balance Scale scores, and clinical descriptive data were collected. Berg Balance Scale and device scores were analyzed with the Rasch model using a repeated-measures approach to examine whether the distribution of exercise scores fitted the Rasch model. Secondly, a person-item map was created to show the hierarchy of exercise difficulty and person ability.

**Results:**

Participants completed a selection of 56 balance exercises (ie, items), which consisted of a combination of various balance tasks and levels (ie, exercises). Using repeated measures, this resulted in a count of 785 observations. Analysis showed strong evidence for unidimensionality of the data. A total of 47 exercises (ie, items) had a sufficiently good fit to the Rasch model. Six items showed underfit, with outfit mean square values above 1.5. One item showed underfit but was kept in the analysis. Three items had negative point-biserial correlations. The final model consisted of 47 exercises, which were provided for persons with low to moderate balance ability.

**Conclusions:**

The VR exercises sufficiently fitted the Rasch model and resulted in a hierarchical order of VR balance exercises for persons with stroke and MS with low to moderate balance ability. In combination with the Berg Balance Scale, the results can guide clinical decision-making in the selection of patient-focused VR balance exercises.

**Trial Registration:**

ClinicalTrials.gov NCT03993275; https://clinicaltrials.gov/ct2/show/NCT03993275

## Introduction

Balance impairments are common in persons with neurological disorders and lead to decreased mobility, increased risk of falling, and accompanied injuries [1,2]. Consequently, balance abilities further degenerate, general mobility reduces, and dependence in activities of daily living increases [3]. This has a significant negative impact on patients’ quality of life; therefore, improvement of balance is key during neurorehabilitation [4,5].

A high treatment dose is important for effective balance rehabilitation; this consists of a high amount of repetitions and challenging exercises [6]. Virtual reality (VR) exergames have gained popularity for delivering VR therapy with a high treatment dose during neurological rehabilitation [7,8]. VR exergames are games that require physical movements to perform exercises in a virtual environment with a therapeutic purpose (eg, to improve strength, balance, or flexibility) [9]. VR is effective for increasing the training dose and delivers good results when used in addition to, or as a partial substitute of, conventional therapy [10]. VR training has been suggested to be effective for improving balance outcomes and gait abilities in persons with neurological disorders, such as multiple sclerosis (MS) or stroke [11-13].

Motivation is an important factor in VR exergames because it influences the duration of play, leading to a higher number of repetitions performed and, thus, increased treatment dose. In previous work by our research group, it was shown that after an initial increase of motivation, over time, motivation in older adults who played exergames decreased in comparison to self-regulated exercises guided by paper forms [14]. An explanation for these results could be found in earlier studies regarding game development [15,16]. Motivation strongly depends on factors like personal calibration of gaming parameters to the player’s motor skills and goals, such as adaption of the range of motion, playing position, or speed of movement. In addition, variation within and between games is important for motivation because insufficient variation reduces focus and physical activity levels [17]. The variation of difficulty is used to keep the player engaged over a prolonged time, but also to progress the task training throughout the rehabilitation process. Progression of task training is important to best stimulate motor learning. This is supported by the challenge point framework, which shows that the difficulty of a task should be adapted to the skill level of the player in order for them to be optimally challenged and to promote learning [18]. Specific parameters that create challenge in balance VR exergames can be speed of play or range of movement during weight shifting [19,20]. From these results, we can presume that in order to keep the patient motivated, it is crucial to challenge the patient by delivering a variation of exercises, whereby the difficulty of the exercise matches the abilities of the patient.

Within robotic upper-extremity therapy, adaption of the difficulty of VR exergames to challenge the player has already been investigated [21]. However, the adaption of the difficulty of VR exergames has been performed based on the performance of the player in specific chosen parameters. This is possible within systems that have similar parameters to adapt difficulty, such as the robotic-guided reaching task from Zimmerli et al [22], who optimized the difficulty of the task by controlling the time that is available for a patient to reach to a given target. However, for therapy programs containing balance exercises this is impossible, as balance exercises are built up out of many different parameters that vary strongly between exercises [23]. Hence, the difficulty adaption of balance exercises needs to be based not on the performance but on the ability of the patient.

A statistical framework that is used in scale development and hereby investigates the difficulty of items and the abilities of persons has already been found in rehabilitation research. La Porta et al [24] investigated the 14 items of the Berg Balance Scale using the Rasch model. The Rasch model was used to investigate the construct of the scale and to order the items of the balance scale from easy and successfully executable by all participants (ie, sitting on a chair) to difficult items that were not executable by all participants (ie, standing on one leg). The model looks at the ability of the person on one hand and at the difficulty of the item on the other hand, and combines both along the continuum of a latent trait [25]. The latent trait is the attribute that all items on the scale have in common and aim to assess. In this study, the abilities of the persons under investigation were those of persons with stroke and MS. The items under investigation were the VR exergames from the MindMotion GO system (MindMaze SA) that are aimed at improving sitting and standing balance. The Rasch model has been widely used to construct and revise measurement instruments and test their psychometric properties [24,26]. The applicability of the Rasch model to data with technology-guided rehabilitation exercises has not been investigated so far and is, thus, an innovative approach to use the model.

To determine if the Rasch model is applicable for delivering a continuum of participant ability and exergame difficulty based on the exergames scores, we evaluated the unidimensionality (ie, if all exergames evaluate the same latent variable) and fit (ie, item fit) of the exergames to the Rasch model. When a continuum of the VR exercises were created, we would investigate whether the exergames deliver enough variation and challenge to the specific participants by establishing the difficulty of each exergame, and we would evaluate whether these difficulty estimates cover the whole spectrum of our participants’ balance abilities.

## Methods

### Recruitment

We aimed to include 30 persons with stroke and 50 persons with MS from the Valens Rehabilitation Clinic in Valens, Switzerland. Patients referred for in-patient rehabilitation were included if they met following criteria: (1) persons with a recent stroke or suffering from MS with an Expanded Disability Status Scale score between 3 and 6.5, meaning patients with moderate to severe disability who need assistance during walking, as confirmed by a neurologist; (2) above 18 years of age; (3) referred for a minimum of 3 weeks of in-patient rehabilitation; (4) reduced balance, based on a Berg Balance Scale score of less than 52 out of 56 points; and (5) signed informed consent. Persons were excluded from the study if they had comorbidities that could interfere with exergame performance, walking ability, and balance (eg, visual or cognitive impairments, psychiatric disorders, and musculoskeletal problems). To include participants with a range of balance deficits that would represent the neurological population, we aimed to include 50% of participants with a Berg Balance Scale score below 45 points (out of 56) and 50% with a Berg Balance Scale score equal to or above 45 points. This study was prospectively registered at ClinicalTrials.gov (NCT03993275).

### Ethics Approval

Ethical approval for this study was obtained from the Ethics Committee of the Sudostschweiz (BASEC [Business Administration System for Ethics Committees] No. 2018-01248), according to the Declaration of Helsinki [27].

### Device and Balance Exergames

**Figure 1:**
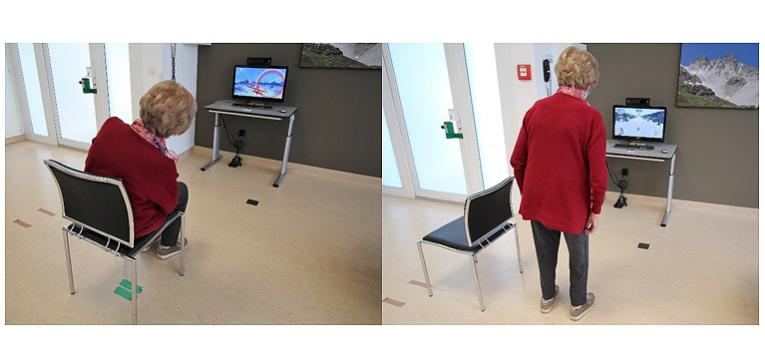
A patient performing exercises with the device under investigation. Exercises in the standing position were performed without aid or physical support, and a chair was placed at arm’s length of the participant for security.

**Figure 2:**
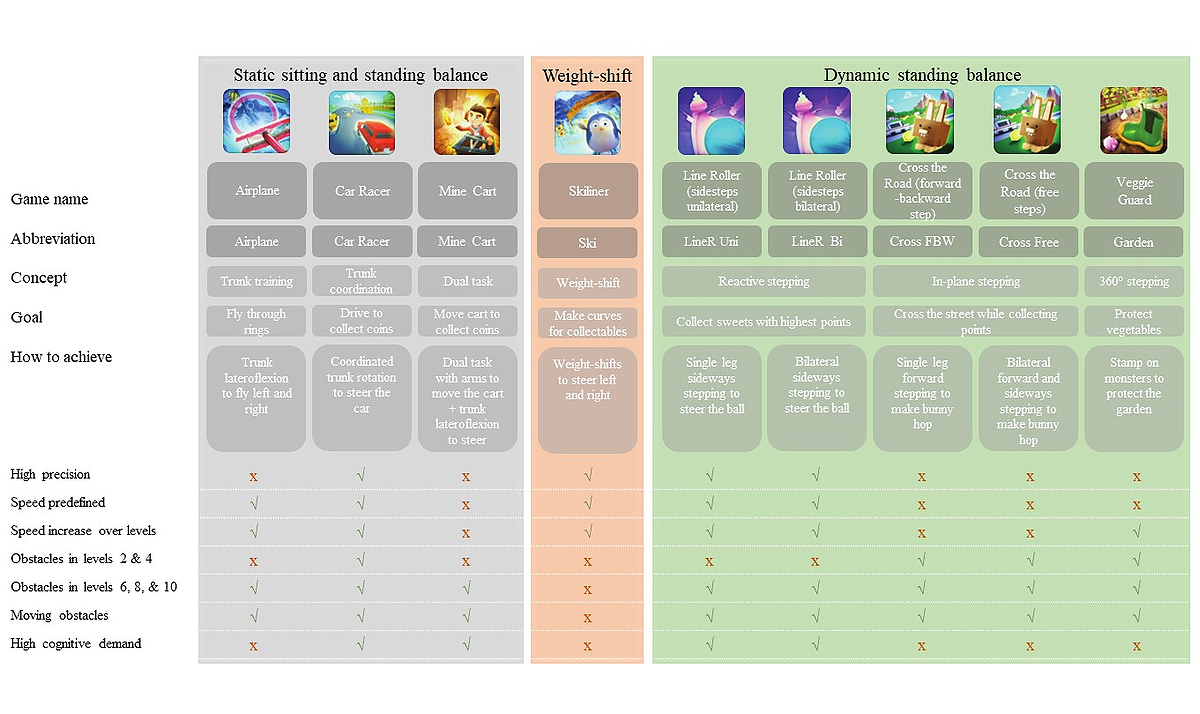
Details of the various exergames. Games are divided into static sitting and standing balance exercises, weight shifting in standing exercises, and dynamic standing balance exercises. Games are described using the following parameters, where a green checkmark indicates the game contains it, while a red X indicates it does not: high precision—high precision of movement is needed in order to steer the avatar well; speed predefined—the speed is constant within the game and cannot be influenced by the player; speed increase over levels—the predefined speed increases with higher level; obstacles in levels 2 & 4 or levels 6, 8, & 10—whether obstacles occur in these levels or not; moving obstacles—obstacles move from left to right or up to down, interfering with the players trajectory; high cognitive demand—the game contains elements such as go-no-go reactions or choices between collectables with different point counts. FBW: forward-backward step.

VR exergames were performed with the MindMotion GO (MindMaze SA). The MindMotion GO is a medical device software system that supports the physical and cognitive rehabilitation of adults and children in rehabilitation centers and of adults at home (Figure 1). The MindMotion GO software is meant to be installed on computers that run the Windows 10 operating system and is used in combination with the Kinect motion sensing device (version 2; Microsoft). The device consists of a screen to visualize the VR exergame and a Kinect camera that traces 25 joints with a rate of 30 frames per second to capture body movements. The camera, together with appropriate motion tracking algorithms, can reliably measure lower-extremity movement in healthy controls and is valid for assessing spatiotemporal gait parameters and kinematic strategies of postural control [28-30]. The software translates human body movements into displayed avatar movements to execute the VR exergame. The software includes a library with rehabilitation exercises for the upper extremities, trunk, and lower extremities. To specifically train trunk control and balance in a seated or standing position, there are nine different VR exergames available. Exergames vary in difficulty, depending on the type of task, playing position, static or dynamic base of support, velocity, and the use of dual tasks and go-no-go responses. Figure 2 details the goals and various difficulty parameters of the different exergames.

In total, nine different games are available, of which three games can be played in both sitting and standing positions (Figure 2, grey) and six games can be played only in the standing position (Figure 2, orange and green). The difficulty increases over 10 levels. To collect sufficient data on all difficulty levels, only levels 2, 4, 6, 8, and 10 were used in the study. This resulted in 60 combinations of VR exergames and levels, henceforth called exercises.

### Design and Data Collection

We implemented a longitudinal observational design with repeated measures of exercise scores and balance ability scores over a duration of 3 weeks among persons with MS and 4 weeks among persons with stroke. After inclusion, descriptive data of trunk control, mobility, gait ability, fatigue, and cognition were collected, and baseline assessments of balance were performed. Once per week during each participant’s general physical therapy session, balance was assessed with the 14-item Berg Balance Scale by an experienced physical therapist. Each item is rated on a scale from 0 to 4, resulting in a maximum score of 56 points. Rasch analyses support the validity of the Berg Balance Scale, and test-retest, interrater reliability, and intrarater reliability are considered adequate to good [24,31].

Participants performed the exercises in a sitting position if their Berg Balance Scale score was lower than 45 points and in the standing and sitting positions if their Berg Balance Scale score was equal to or above 45 points. Exercises were explained and participants completed a practice trial at the middle level of difficulty for 30 seconds. This result was not used for analysis. Participants played at a lower level if the score of the practice trial was below 50% and at a higher level when 50% or higher. Participants completed each exercise for 2 minutes. Exercise scores were used for analysis. Participants rated exercise difficulty on a 6-point Likert scale, ranging from 0 (“very easy”) to 5 (“unable to perform”). Participants’ ratings of exercise difficulty were used to adapt exercise choice. During each therapy session, participants completed six different exercises. A variation of exercises was completed throughout the therapy sessions to make sure that each participant completed as many different exercises as possible, while considering the safety of the participant. Training was guided by an assistant under the supervision of an experienced physical therapist who specialized in robotics therapy.

### Statistical Analysis

#### Sample Size

A minimum sample size of 150 observations was needed to achieve stable item calibration within ±½ logits and 99% confidence [32]. To reach this number of observations but still be able to perform a feasible study design with equality between groups, persons with MS were included in the study for 3 weeks, whereas persons with stroke were included for 4 weeks. Multiple observations were accounted for according to the following rules: (1) at the start of the week, the first session was accounted for as observation 1; (2) as soon as a participant completed an exercise twice within the same week, this was a new observation; and (3) the exergame scores were analyzed with the scores on the Berg Balance Scale of that week. Thus, this resulted in multiple repeated observations of the same participant, creating data that were partially independent. However, Rasch analysis can confidently be performed with this type of data [33].

#### Analysis

Descriptive statistics were used to describe the study participants. The main analysis was performed using the Rasch analysis. The Rasch model is a mathematical model that delivers the expected response probability that shows how likely it is that a person with a certain ability (ie, number of correct test items) can perform an item of a certain difficulty (ie, number of persons who succeeded on the item) in a correct manner. The item level represents the difficulty of an item, whereby the difficulty of items range from very easy to very difficult. The person level represents the ability of the person that undertakes the item. Both are expressed in the natural log of an odds ratio (ie, logits) [34]. The higher the logit value, the more difficult the item and the higher the ability of the person [35]. The probability of success depends on the difference between the difficulty of the item and the ability of the person [25]. The advantage is that the scale under investigation becomes an interval scale and, thus, an improvement of one logit has the same value among the continuum of the scale independent of the difficulty of the item. Therefore, the use of the Rasch model enables the choice of the right exercise for the person’s ability so that they are able to pass it without it being too easy, thus creating enough challenge for the patient without developing frustration.

We used Winsteps (version 4.5.5) to evaluate the fit of the data to the Rasch model [36]. The partial credit model was used because the structure of game scores is not comparable across the different exercises [37]. To use the Rasch model in a correct manner, unidimensionality and item fit were investigated, which show if the various exergames all train the same latent trait. The main concepts of the Rasch model are explained in the following paragraphs.

#### Unidimensionality

Unidimensionality is given when all items (ie, exercises) under investigation measure only one single latent variable [25]. Unidimensionality was investigated with the principal component analysis of the residuals [38]. An eigenvalue of greater than 2 was indicative for a potential secondary dimension [39]. Hereby, we consider the amount of raw unexplained variance within the contrast for our large number of items. A contrast plot was used to evaluate unidimensionality while looking closer at deviating items or patterns. The disattenuated correlation coefficients were assessed to see if the data between possible different dimensions are related and measure the same latent trait. Values below 0.3 were considered problematic, and values above 0.7 that are close to 1 would indicate that items from different clusters measure the same trait [40].

#### Point-Biserial Correlation

In Rasch analysis, the item correlations are an immediate check that the response level makes sense, meaning that with increasing ability, item (ie, exercise) scores also increase. If the observed correlation is negative, the response collection was wrong. This can be due to several reasons (eg, a reversed survey item was overlooked). These items were removed from the final model.

#### Item Fit

Fit statistics quantify the difference between the theoretical expectation based on the Rasch model and the actual item performance of the raw data, thus indicating how good the data fitted the Rasch model. Larger residuals denote an item that does not fit the model. Item fit was investigated with the fit statistics, whereby outfit mean square values between 0.5 and 1.5 are productive for measurement [41]. Values above 1.5 were classified as showing underfit; this means that there was more noise in the performance of the item and, therefore, this item cannot be used to make adequate predictions [25]. Items that showed misfit, without a clinical reasonable explanation, were removed from the analysis.

Values below 0.5 were classified as showing overfit; this means that multiple items were strongly interdependent and, thus, redundant for measurement. This derives, for instance, from items that are strongly interrelated by nature (eg, the development in rehabilitation of standing, stepping, and walking); therefore, responses are too predictable from other exercises. In the case of scale development, removing redundant items increases efficiency without reducing precision. However, as we were not developing a measurement instrument, but a construct of exercises, redundant items could be left in the model, as they increase variation in the exercise program for participants.

#### Ordering of the Item Thresholds

Thresholds represent the point where the chance of having a score of 0 or 1, or 1 or 2, are equal. The item threshold shows if the order of scores for a certain exercise is logical in a way that a participant with the ability to succeed at the task at hand can succeed when the items undertaken get more difficult. As persons advance and their skills get better, it is important that the categories of scores represent advancing levels of the construct under investigation.

#### Distribution of Responses: Person-Item Map

The hierarchy of the exercise difficulty and participant’s abilities is shown in a person-item map. In this map, the easiest items are on the lower right side of the map, and the participant with the lowest ability is displayed at the lower left side of the map. The map allows visual inspection of whether the range of exercises targets the range of participants’ abilities.

#### Differential Item Functioning

Differential item functioning (DIF) displays the difference in difficulty of the items for persons with stroke and persons with MS. This is assessed through comparison of both groups, whereby persons with stroke and persons with MS should show the same ability on an item, that is, the same logit value (ie, same DIF measure).

## Results

### Overview

Descriptions of the persons with stroke and persons with MS can be found in Table 1. A total of 32 persons with stroke and 52 persons with MS were recruited. A total of 30 persons with stroke and 51 persons with MS completed the data collection step and were included in the analysis; 2 persons with stroke and 1 person with MS dropped out after baseline measurements. Severity of balance disorder as measured by the Berg Balance Scale was comparable between pathology groups (*P*=.35). A total of 785 observations were recorded to reach stable item calibration. Adherence to the training program was very good (99%). No serious adverse events occurred during training with the device. Two falls from the chair without injuries or other consequences were recorded during training; this is comparable to conventional balance training at the limits of balance. Four out of 60 exercises were not included in the analysis because they were performed only two or four times and, thus, not enough data were available (Tables 2 and 3). In total, 56 exercises were included in the analysis, together with the 14 Berg Balance Scale items. The analysis consisted of two rounds to develop the final model (Table 2). The final model included 47 exercises (Table 4).

**Table 1:** Descriptions and clinical measures of the participants.

Characteristic		Participants with stroke (N=30)	Participants with multiple sclerosis (N=51)	
Age (years), median (IQR)		64.50 (55.50-76.75)	55.00 (46.00-60.00)	
**Gender, n (%)**				
	Male	22 (73)		13 (25)
	Female	8 (27)		38 (75)
**Type of stroke, n (%)**				
	Ischemic	27 (90)		N/A^a^
	Hemorrhagic	3 (10)		N/A
**Type of multiple sclerosis, n (%)**				
	Primary-progressive multiple sclerosis	N/A		13 (25)
	Secondary-progressive multiple sclerosis	N/A		19 (37)
	Relapse-remitting multiple sclerosis	N/A		19 (37)
**Hemiparetic or weaker body side, n (%)**				
	Left	12 (40)		26 (51)
	Right	16 (53)		24 (47)
	Bilateral	2 (7)		1 (2)
Time poststroke (days), median (IQR)		14.00 (11.25-20.75)	N/A	
Time since multiple sclerosis diagnosis (years), median (IQR)		N/A	16.0 (10.00-20.50)	
**Functional Ambulation Category, n (%)^b^**				
	0-2	13 (43)		0 (0)
	3-5	17 (57)		51 (100)
Berg Balance Scale score, median (IQR)^c^		41.00 (26.00-47.00)	44.00 (33.50-47.00)	
Trunk Impairment Scale score, median (IQR)^d^		16.00 (13.00-18.75)	17.00 (14.50-18.00)	
Dynamic Gait Index score, median (IQR)^e^		11.00 (0.00-17.00)	13.00 (8.00-17.00)	
Timed Up and Go test time (seconds), median (IQR)^f^		21.00 (12.00-33.00)	16.00 (11.00-28.00)	
Montreal Cognitive Assessment score, median (IQR)^g^		23.00 (20.00-24.75)	25.00 (23.00-27.00)	

^a^N/A: not applicable; this measure does not apply to this group of participants.

^b^Functional ambulation scores range from 0 (a patient cannot walk or needs help from two or more persons) to 5 (a person can walk anywhere independently).

^c^The Berg Balance Scale has a maximum score of 56 points, with more points meaning better balance.

^d^Trunk Impairment Scale scores range from 0 to 23, with higher scores meaning better trunk function.

^e^Each item of the Dynamic Gait Index is scored on a scale of 0 (severe impairment) to 3 (normal performance); the maximum total score is 24.

^f^The Timed up and Go Test measures the time to stand up from a chair, walk 3 meters, turn, walk back, and sit down again; the lower the duration of this assessment, the better.

^g^The Montreal Cognitive Assessment has a maximum score of 30 points.

**Table 2:** Overview of the performed steps in the Rasch analysis.

Analysis number	Description of the analysis	Observations, n	Items, n	Decisions
1	Initial analysis	785	70	Exclude 3 items based on negative point-biserial correlations Exclude 6 items based on item misfit
2	Final analysis	785	61	Although misfit, leave Car Racer level 10 in the sitting position item in the analysis

**Table 3:** Reasons for deleting items from Rasch analysis and deleted items.

Reason for deletion	Deleted items (levels)
The exercise was performed only two to eight times; thus, not enough data were available.	Cross the Road: free steps (2) Veggie Guard (2) Veggie Guard (10) Line Roller: sidesteps unilateral (10)
The exercise resulted in negative point-biserial correlations.	Cross the Road: free steps (4) Veggie Guard (4) Line Roller: sidesteps bilateral (10)
The exercise was excluded because of underfit.	Airplane (4) Airplane (8) Car Racer (2)^a^ Cross the Road: forward-backward step (10) Veggie Guard (6) Mine Cart (2)^a^

^a^These exercises were performed in a seated position.

**Table 4:** Items included in the final model in order of decreasing difficulty.

Item (level)^a^	Item parameter			Fit statistics		
	Measure (logits)	Model SE	Infit mean square		Outfit mean square	Point-biserial correlation
Line Roller: sidesteps bilateral (10)	4.62	0.53	0.42		0.53	0.72
Line Roller: sidesteps unilateral (8)	4.55	0.50	0.50		0.53	0.34
Car Racer (8)	4.10	0.15	0.61		0.71	0.77
Car Racer (10)	3.98	0.23	0.49		0.56	0.78
Car Racer (10)^b^	3.86	0.13	0.56		1.56	0.74
Line Roller: sidesteps bilateral (6)	3.10	0.31	0.45		0.50	0.85
Car Racer (8)^b^	2.81	0.09	0.63		0.87	0.74
Line Roller: sidesteps unilateral (6)	2.74	0.29	0.32		0.33	0.92
Line Roller: sidesteps unilateral (4)	2.72	0.23	0.70		0.70	0.68
Line Roller: sidesteps bilateral (4)	2.64	0.25	0.78		0.86	0.65
Berg Balance Scale item 14	2.49	0.09	1.28		1.31	0.72
Line Roller: sidesteps unilateral (2)	2.29	0.25	0.90		0.98	0.58
Berg Balance Scale item 12	1.93	0.08	1.37		1.17	0.78
Line Roller: sidesteps bilateral (2)	1.93	0.31	0.56		0.58	0.49
Cross the Road: free steps (10)	1.54	0.48	0.44		0.46	0.94
Veggie Guard (8)	1.53	0.36	0.48		0.50	0.69
Berg Balance Scale item 13	1.47	0.08	1.04		1.03	0.78
Berg Balance Scale item 11	1.32	0.08	0.90		0.87	0.83
Car Racer (4)	1.10	0.17	0.79		0.79	0.66
Car Racer (6)	0.78	0.14	1.00		1.05	0.63
Cross the Road: free steps (6)	0.69	0.54	0.97		0.90	0.72
Skiliner (5)	0.62	0.17	0.83		1.07	0.66
Mine Cart (8)	0.50	0.23	1.08		0.99	0.62
Cross the Road: free steps (8)	0.38	0.63	0.33		0.24	0.88
Skiliner (4)	0.37	0.14	0.81		0.96	0.60
Mine Cart (10)	0.20	0.38	0.79		1.21	0.44
Skiliner (3)	0.19	0.19	0.91		0.80	0.66
Berg Balance Scale item 7	0.07	0.09	1.18		0.97	0.80
Car Racer (4)^b^	0.02	0.11	1.11		1.34	0.65
Car Racer (6)^b^	–0.14	0.09	0.98		0.95	0.73
Cross the Road: forward-backward step (6)	–0.19	0.28	1.53		1.20	0.68
Berg Balance Scale item 8	–0.22	0.10	1.07		1.43	0.74
Skiliner (2)	–0.33	0.25	0.76		0.70	0.72
Mine Cart (10)^b^	–0.34	0.13	1.17		1.20	0.56
Cross the Road: forward-backward step (4)	–0.35	0.35	0.85		0.83	0.66
Berg Balance Scale item 10	–0.59	0.10	0.89		0.83	0.77
Berg Balance Scale item 9	–0.73	0.11	1.12		0.74	0.77
Airplane (2)	–0.94	0.56	1.83		1.34	0.59
Cross the Road: forward-backward step (8)	–0.95	0.39	1.16		0.76	0.47
Mine Cart (2)	–1.17	0.54	0.71		0.51	0.71
Mine Cart (6)	–1.27	0.27	0.76		0.75	0.51
Berg Balance Scale item 6	–1.43	0.12	0.87		0.78	0.73
Mine Cart (8)^b^	–1.45	0.15	1.33		1.17	0.58
Berg Balance Scale item 5	–1.92	0.13	1.19		0.75	0.70
Cross the Road: forward-backward step (2)	–1.94	0.65	1.18		1.08	0.42
Car Racer (2)	–1.98	0.54	0.97		0.75	0.61
Skiliner (1)	–2.06	1.06	1.08		0.99	0.14
Airplane (10)	–2.07	0.37	1.12		1.36	0.28
Mine Cart (4)	–2.17	0.41	0.68		0.27	0.70
Berg Balance Scale item 1	–2.19	0.14	0.86		0.77	0.70
Berg Balance Scale item 4	–2.29	0.14	1.05		0.68	0.66
Mine Cart (4)^b^	–2.45	0.17	1.27		1.43	0.54
Airplane (10)^b^	–2.61	0.18	1.37		1.25	0.34
Berg Balance Scale item 2	–2.68	0.16	1.03		0.33	0.64
Airplane (4)^b^	–2.70	0.24	1.47		1.13	0.59
Mine Cart (6)^b^	–2.80	0.18	1.28		1.24	0.47
Airplane (8)^b^	–3.33	0.22	1.47		1.18	0.35
Airplane (6)	–3.38	0.62	0.80		0.51	0.39
Airplane (2)^b^	–3.87	0.45	1.09		0.49	0.63
Airplane (6)^b^	–4.03	0.25	1.31		0.80	0.46
Berg Balance Scale item 3	–8.97	1.83	mm^c^		mm	0.00

^a^Exergames are described in Figure 2.

^b^These exercises were performed in a seated position.

^c^mm: minimum measure.

### Unidimensionality

As part of the partial credit model, the principal component analysis of the residuals showed that the first residual contrast had an eigenvalue of 2.36, indicating a possible secondary dimension with the strength of 2.3 items. This was close to the predefined threshold for unidimensionality of 2 eigenvalues. Given the large number of analyzed exercises (n=61), a possible secondary dimension with the size of 2.3 items is very small. In addition, the total unexplained variance in the first contrast was only 3.9% (of the total raw unexplained variance in the analysis). Visual exploration of the contrast plot of standardized residuals revealed that the device exercises had similar factor loadings and were grouped together. The Berg Balance Scale items were within the same dimension; however, eight of them covered a different facet of balance. All disattenuated correlations were close to 1, indicating that all items within the dimension measured the same trait. Therefore, it can be concluded that unidimensionality was adequate.

### Point-Biserial Correlation

In the first round of the analysis, three point-biserial correlations were negative, indicating that item scores were negatively correlated with balance ability. Consideration of the items from a clinical point of view resulted in the exclusion of these items in the final model (Table 3; Cross the Road: free steps, level 4; Veggie Guard, level 4; Line Roller: sidesteps bilateral, level 8). In the final model, there were no negative point-biserial correlations, indicating that all items worked in the intended way.

### Item Fit

**Figure 3:**
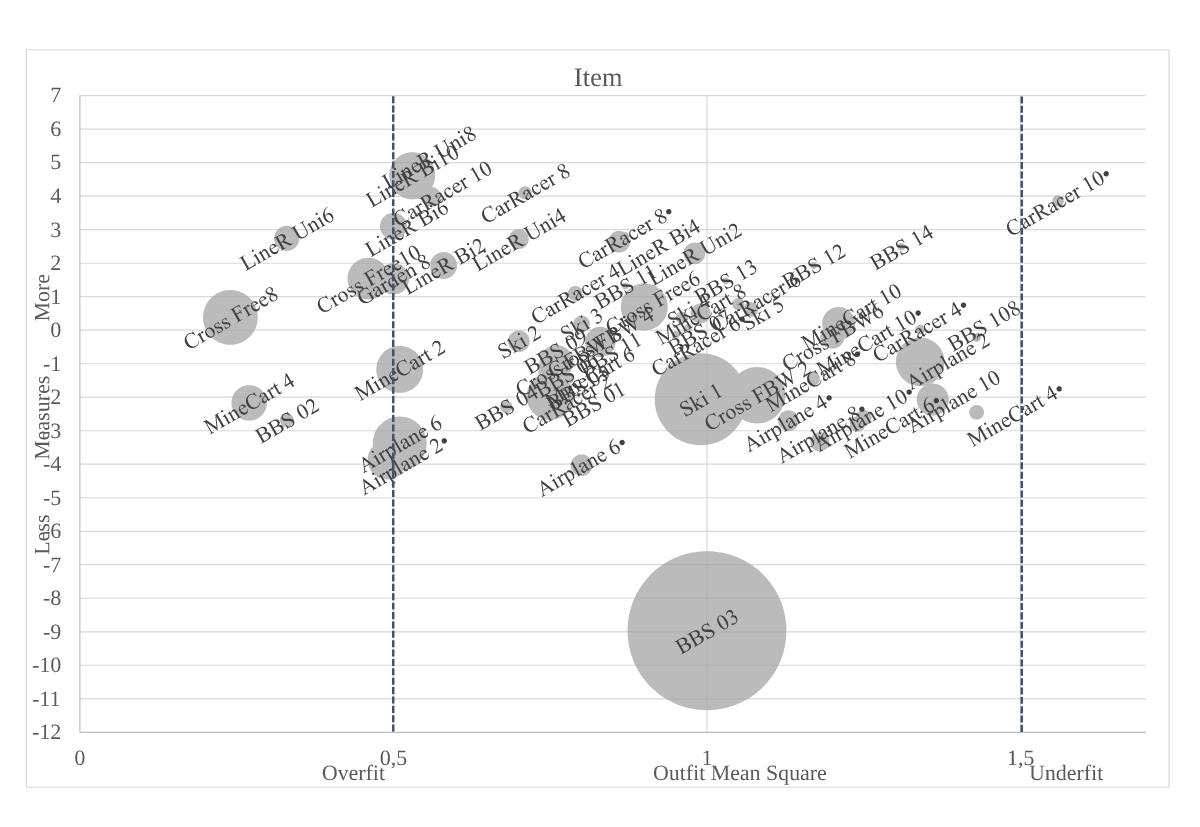
Bubble plot with the outfit mean square statistics. Game descriptions can be found in Figure 2. Outfit mean square values should range from 0.5 to 1.5; items below 0.5 show overfit and items above 1.5 show underfit. The size of the bubble shows the model SE. The number beside each exergame represents the exergame level, and the ● denotes the games that are performed in a seated position. The number beside BBS represents the scale item number. BBS: Berg Balance Scale; Cross FBW: Cross the Road (forward-backward step); Cross Free: Cross the Road (free steps); Garden: Veggie Guard; LineR Bi: Line Roller (bilateral); LineR Uni: Line Roller (unilateral); Ski: Skiline.

Based on outfit mean square statistics, values above 1.5 appointed the items that showed underfit and, thus, did not fit the model. This resulted in the deletion of following items after the first round of analysis (Table 3): Airplane, level 4, in the standing position (1.79); Airplane, level 8, in the standing position (2.12); Car Racer level 2, in the sitting position (1.77); Cross the Road, forward-backward step, level 10 (1.64); Veggie Guard, level 6 (3.53); and Mine Cart, level 2, in the sitting position (1.95). Various items showed values below 0.5, indicating overfit and, thus, making items redundant. However, in our study it was not the goal to delete redundant items, but to find multiple well-targeted items to deliver variation to our participants. Thus, we decided to keep these items in the model. In the final round of the analysis, only one item (ie, Car Racer, level 10, in the sitting position) showed slight underfit, with a value of 1.56 (Figure 3). We set 1.5 as our threshold in the first round of the analysis. However, Wright and Linacre [41] argue that a threshold of 1.7 is acceptable for clinical observations. Because the aforementioned item only slightly deviated from the statistical rule and had its own logit value, we did not delete this item from the analysis.

### Ordering of the Item Thresholds

The ordering of the item thresholds was acceptable. For 38 items, the whole score range was not used and, therefore, not all thresholds were available. For nine items, the thresholds were not in the correct order, meaning the score did not increase stepwise with increasing ability.

### Distribution of Responses: Person-Item Map

**Figure 4:**
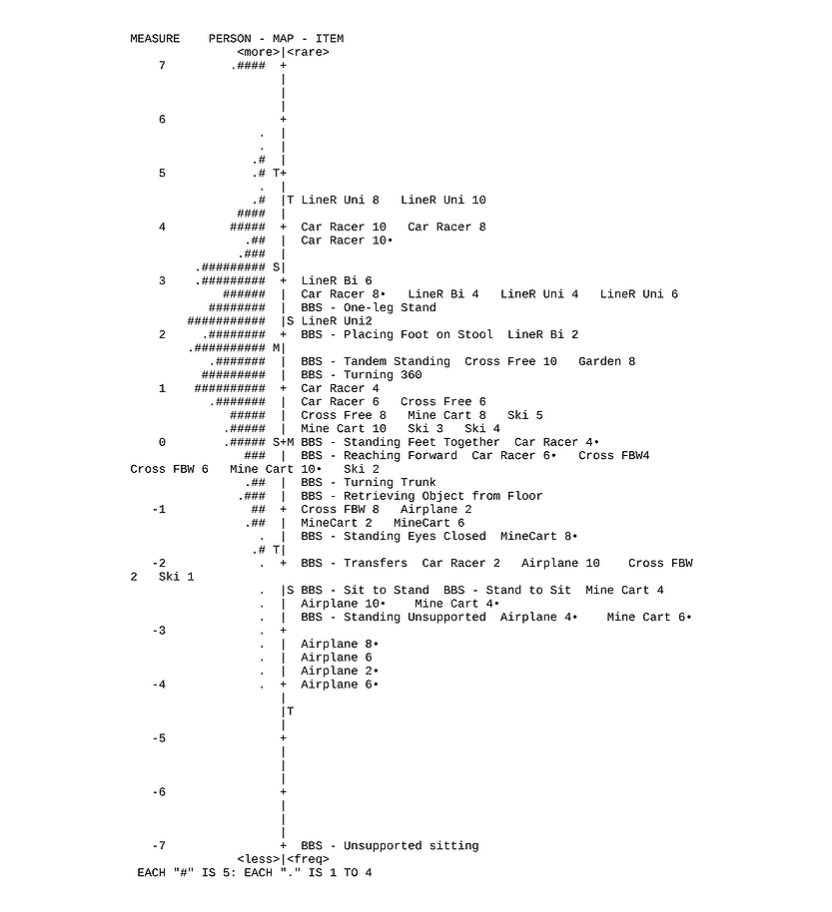
Person-item map. This map shows the person ability and item difficulty in one scale expressed in logit values. Person ability is shown on the left side, with the lowest person ability at the bottom and highest at the top. Item difficulty is shown on the right side, whereby items are organized from least difficult at the bottom to most difficult at the top. The number beside each exergame represents the exergame level, and the ● denotes the games that are performed in a seated position. The number beside BBS represents the scale item number. BBS: Berg Balance Scale; Cross FBW: Cross the Road (forward-backward step); Cross Free: Cross the Road (free steps); Garden: Veggie Guard; LineR Bi: Line Roller (bilateral); LineR Uni: Line Roller (unilateral); M: mean; S: one SD; Ski: Skiline; T: two SD.

Figure 4 shows the final person-item map; the ability of the participant is represented on the left side and the difficulty of the items is represented on the right side of the figure. Person ability ranged from –4.0 logits to 7.0 logits, and item difficulty ranged from –4.0 logits to 4.75 logits. In general, it is seen that for participants with lower to moderate balance abilities, a great variation of exercises exists. The variation of exercises is lower for participants with higher balance abilities, especially for participants with very high balance abilities (eg, for logits of 4.5 and higher, there are no matching items available).

### Pairwise DIF Contrast

The pairwise DIF contrast between persons with MS and persons with stroke showed a significant difference in difficulty in 15 out of 61 items. Of these items, only 10 showed a substantial difference of 0.5 logits, which presents a clinically noticeable difference between the groups. Of these, 7 items were exergames and 3 items were Berg Balance Scale items.

## Discussion

### Principal Findings

Overall, the results of our study showed adequate unidimensionality and good fit statistics of the exergame scores to the Rasch model. The resulting person-item map showed that the exergames covered the abilities of persons with neurological disorders with low to moderate balance ability and delivered enough variation of exercises, especially for persons with moderate abilities.

The VR exercises and many of the Berg Balance Scale items showed adequate unidimensionality. However, eight Berg Balance Scale items were at the limit of the first dimension and measured a different aspect of balance than the other items. The difference arose because the Berg Balance Scale measures static and basic balance over short time periods, whereas the exercises measure static, dynamic, and reactive balance over longer time periods of up to 2 minutes. Consequently, both of these measure balance but, as indicated by the analysis, they measure a different aspect of balance.

Fit statistics showed that 47 out of 60 exercises fitted well to the Rasch model. Three items had to be deleted from the final model because of negative point-biserial correlations. This could be due to wrongful score calculations, in combination with other game parameters that have a considerable influence on the score calculation, such as obstacles to avoid or simultaneous collection of collectables. As a consequence, the score did not reflect the participant’s ability (eg, the score goes up even if the ability of the participant does not increase).

Six items were deleted after the initial analysis because of underfit; thus, these items included too much noise and did not fit sufficiently to the Rasch model. The corresponding items showed a large IQR in scores. Clinically, it was noted that certain exercises were too easy, such as Car Racer, level 2 (in the sitting position)*.* Consequently, participants with all abilities were often distracted and reached various scores that did not seem to match with their ability. In the final analysis, Car Racer, level 10 (in the sitting position), showed slight misfit. We used 1.5 as a stricter cutoff value; however, Wright and Linacre [41] argue that a threshold of 1.7 is also acceptable for clinical observations. Since the exercise was in the top range of difficulty, had its own logit value of difficulty, and was appreciated by therapists to challenge the participant in a seated position, it was deliberated among experts to keep the exercise in the analysis. Usually, the goal of a Rasch analysis, when used for scale development, is to delete items with misfit or that are redundant, in order to make the scale leaner and less time-consuming. However, in this study, we explored the data for challenging exercises that fit the model in a proper manner. Next to that, redundancy was not an issue in our study, as variation of exercises was important for keeping the motivation of the participant as high as possible.

In the second part of the analysis, the person-item map was constructed to show the difficulty estimate of items and the ability of the participants. The device under investigation provided mainly VR exercises for the severely to moderately affected participants, with most variation for the moderately affected participants. The person-item map showed few patients with very high balance ability in the top, for whom no suitable exercises were available. However, it should be noted that not all exercises were performed, as therapists did not use four exercises because they were considered unsafe for the participant. Regarding the more difficult VR exercises, it was seen that the maximum score was not achieved. This shows us that possibilities were available for this patient group, but these were not clinically used. Therefore, the use of a harness in higher-level balance exercises is recommended, in order to adequately and safely challenge the patient on the limits of their balance capabilities.

Research into VR-based adaptive training mainly investigates how to challenge a patient within a single exercise by adapting parameters, such as accuracy, speed, and amplitude of movement [42]. By investigating only a single exercise, the importance of variation as well as progression of tasks is not addressed. The performed analysis provides an opportunity for the development of training programs in neurological rehabilitation. In sports injury rehabilitation, many protocols exist for graded rehabilitation with variation of exercises and intensity of training to optimally challenge the patient and work toward recovery. In neurological rehabilitation, such clinical protocols are scarce. A study by Wüest et al [43] is one of the few to discuss the theoretical design considerations for an exergame-based rehabilitation program that aims to improve walking in the stroke population. The model showed a clear progression in relearning how to walk, whereby exercises progressed from a stable body position with a stable environmental context to exercises with body transport and in-motion environmental context. Our clinical data–based model showed similarity with regard to the sequencing of exercises in the standing position within the model, whereby a progression was seen from weight-shifting activities to stepping activities and changing environmental contexts throughout the different exercises. Through the performed analysis, an idea was formulated toward a protocol of training balance ability using technologies in the neurological population and how to increase difficulty and, thus, challenge, while progressing through the different rehabilitation VR exercises. This model is further supported by the clinically established Berg Balance Scale that validates the VR exercises and delivers a clinically established starting point from which to choose VR exercises in daily practice [44]. Further use of the model in similar study designs and data types could show the potential of the Rasch model in creating challenging and adapted training programs with various technologies in specific patient populations.

The analysis faced some limitations. From a statistical point of view, the difficulty estimates of the various VR exercises lay in certain items very close together, meaning that exercises were almost equally difficult. The deduction of points for obstacle hits was too large and, thus, had a rather big influence on the exercise final score. Therefore, the person-item map should be interpreted with caution, and clinicians should always integrate their clinical opinion about the safety of the patient when choosing exercises. Therefore, the person-item map is a support rather than a fixed guideline. For the collection of the large amount of data, the repeated-measures model was used. This means that scores from one participant were used as independent scores to establish a minimum number of observations. In this study, an observation was created for each training week, combining one Berg Balance Scale item with VR exercises until a double-played exercise was noted. This resulted in the second observation of the week, and so on. Based on clinical experience, we presumed that participants’ skills stay rather constant throughout the week and, thus, combining scores in this manner was feasible. The choice for this method was based on the clinical character of the study. Not every participant could play all exercises because of severely affected motor function and increased risk of falling. Next to that, there were not enough resources available to meet the high number of single observations needed for stable item calibration. A previous study by Anselmi et al [45] confirmed that repeated measurements using a health questionnaire are feasible. Therefore, we decided to use the repeated-measures model, which resulted in a good fit to the Rasch model. Data were collected from a broad neurological population with severely affected to good balance abilities. We included persons with MS and stroke, as both pathologies are often seen within the neurorehabilitation setting, and these persons suffer from similar impairments with regard to functional balance and trunk control. Therefore, results are applicable to a broad neurological population. Future research could also include healthy subjects with whom to compare results.

The person-item map can stimulate integration of various VR exercises, as it supports the clinician in the decision-making of which exercise is appropriate for which ability as well as in deciding how to continue challenging the patient by using VR and following the continuum of exercises. In this way, this would keep the player challenged and deliver variation in order to keep the player motivated over time and, thus, increase effectiveness of treatment. Future work could focus on the implementation of the person-item map in the clinical field and the feasibility of such within the device. In a second step, in line with the systematic literature review of Zahabi et al [42], the effectiveness of adaptive VR-based training should be investigated in studies with a large sample size with long-term follow-up, in order to assess the transfer of learned skills to activities of daily living.

### Conclusions

The Rasch model was shown to be applicable for creating a continuum of participant ability and exergame difficulty based on VR exergame scores. Unidimensionality of the data was adequate and 47 items showed a good fit to the model. A continuum of exercises was created, whereby it was seen that persons with low and moderate balance ability could be challenged well with the exercises and most variation was available for persons with moderate balance abilities. With this continuum, therapists are supported to choose the correct exercise that delivers the optimal challenge according to the player’s ability. These findings hold promise for the application of the Rasch model within the future development of challenging and tailor-made VR exercise programs for persons with MS and stroke. In future work, the implementation of such a program into clinical practice could be explored as well as the extension of use of the Rasch model for data from different rehabilitation technologies and patient populations.

## Abbreviations

BASEC: Business Administration System for Ethics Committees
DIF: differential item functioning
MS: multiple sclerosis
VR: virtual reality
